# Supplementary material for: Distinct receptor binding domain IgG thresholds predict protective host immunity across SARS-CoV-2 variants and time
Source: Nat Commun. 2023 Nov 2;14:7015. doi: 10.1038/s41467-023-42717-1 (PMC10622572; doi:10.1038/s41467-023-42717-1)
Supplement: Supplementary file 1 — Supplementary information [file 41467_2023_42717_MOESM1_ESM.pdf]

Supplementary Information

The All Ireland Infectious Diseases Cohort Study Investigators:

Mater Misericordiae University Hospital: A. Cotter, E. Muldoon, G. Sheehan, T. McGinty, JS. Lambert, S. Green, K. Leamy. St Vincent's University Hospital: G. Kenny, K. McCann, R. McCann, C. O'Broin, S. Waqas, S. Savinelli, E. Feeney, PWG. Mallon. CEPHR: A. Garcia Leon, S. Miles, D. Alalwan, R. Negi. Beaumont Hospital: E. de Barra, S. McConkey, K. Hurley, I. Sulaiman. University College Cork: M. Horgan, C. Sadlier, J. Eustace. University College Dublin: C. Kelly, T. Bracken. Sligo University Hospital: B. Whelan, Our Lady of Lourdes Hospital: J Low. Wexford General Hospital: O Yousif. University Hospital Galway: B. McNicholas. St Luke's Hospital Kilkenny: G. Courtney. Children's Health Ireland: P. Gavin.

VACCELERATE - EU-COVAT-1 Part A Study Group:

University of Cologne: Julia M. Neuhann, Jannik Stemler, Ullrich Bethe, Sarah Heringer. Jon Salmanton-García, Lea Tischmann, Arnd Cüppers, Jan Grothe, Philipp Koehler, Oliver A. Cornely. Hospital La Paz: Antonio J. Carcas, Jesús Frías-Iniesta. Hacettepe University School of Medicine: Murat Akova, CEPHR: Alejandro Garcia Leon, Patrick Mallon, Riya Negi, Colette Gaillard, Gurvin Saini. University of Antwerp: Christine Lammens, An Hotterbeekx, Katherine Loens, Surbhi Malhotra-Kumar, Herman Goossens, Samir Kumar-Singh, Franz König, Lusine Yeghiazaryan, Martin Posch.

Supplementary Figure 1 – Flow Cytometry Gating Strategy for Neutralisation Assay

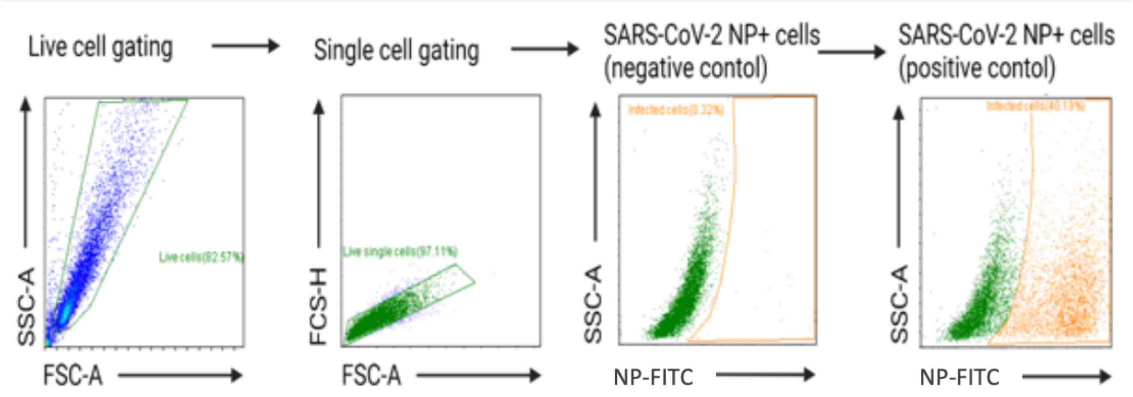

Cells were gated against Forward (FSC) and Side-Scatter (SSC) to exclude debris from intact cells, then single cells were gated based on linearity between Area (A) and Height (H). Infected cells (NP+) were gated against the negative controls (Infection Medium alone). Analysis was performed using CytExpert software (version 2.4.0.28, Beckman Coulter).

Supplementary Figure 2 – Gating Strategy for SARS-CoV-2 specific T cell response

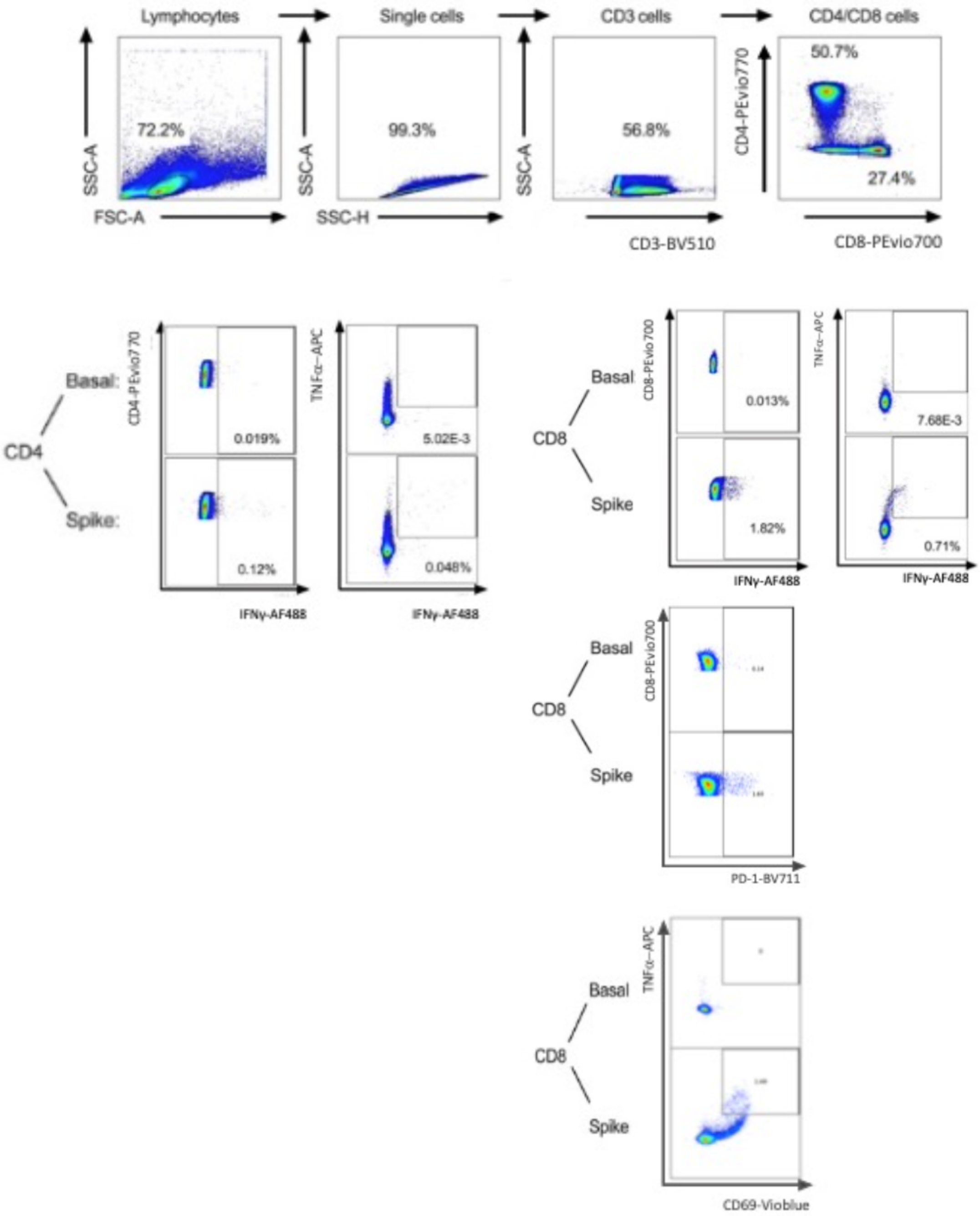

Representative flow cytometry gating strategy for detection of IFNγ+ and TNFα+ responses in CD4+ and CD8+ T cells, and PD-1+ and CD69+ TNFα+ CD8+ T cells. Basal represents the unstimulated control.

Supplementary Figure 3 – Correlation between RBD and NT50 in the VACCELERATE EU-COVAT-1 AGED Part A Cohort

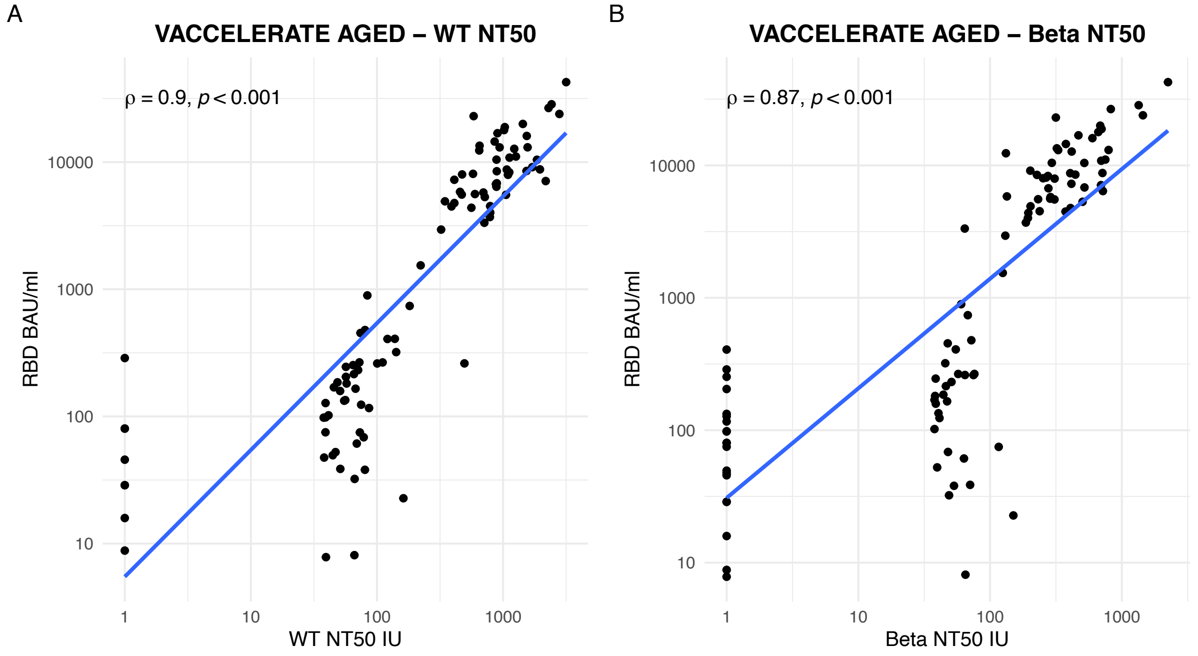

Legend: Two tailed Spearman correlation between A) RBD and WT-B.1.177.18 (exact p value <2.2e-16) NT50 B) RBD and Beta NT50 (exact p value <2.2e-16)

Supplemental Table 1 – Two tailed Spearman correlation of all antigens with WT-B neutralising capacity in all samples and >30 days from symptom onset only

	All samples		>30 days from symptom onset	
	rho	P value	rho	P value
RBD	0.81	<2.2e-16	0.85	<2.2e-16
S1	0.80	<2.2e-16	0.84	<2.2e-16
S2	0.72	<2.2e-16	0.76	<2.2e-16
N	0.74	<2.2e-16	0.72	<2.2e-16

Supplemental Table 2 – Sensitivity and specificity of each antigen in predicting a WT-B NT50 of <1000 IU in all samples and in >30 days from symptom onset

	All samples		>30 days from symptom onset	
	**Sensitivity (95% CI)**	**Specificity (95% CI)**	**Sensitivity (95% CI)**	**Specificity (95% CI)**
**RBD**	83% (76-89%)	73% (56-87%)	82% (74-88%)	80% (56-94%)
**S1**	87% (80-91%)	59% (41-75%)	87% (79-92%)	58% (33-80%)
**S2**	72% (65-79%)	65% (46-80%)	72% (63-79%)	74% (49-91%)
**N**	68% (61-76%)	85% (69-95%)	67% (58-75%)	100% (82-100%)

Supplemental Table 3 – Two tailed Spearman correlation between WT-B NT50 and spike specific T cell responses

**Spike specific response**	**Spearman rho**	**P value**
**CD4+ IFNγ+**	0.21	0.24
**CD4+ TNFα+ IFNγ+**	-0.02	0.92
**CD4+ PD-1+**	0.03	0.86
**CD8+ IFNγ+**	0.09	0.61
**CD8+ TNFα+ IFNγ+**	0.08	0.66
**CD8+ CD69+ TNFα+**	0.27	0.13

Supplemental Table 4 – Spike specific responses in those with an RBD IgG above or below 456 BAU/ml

Spike specific response	>456 BAU/ml	<456 BAU/ml	P value
**CD4+ IFNγ+**	0.065 (0.02-0.1)	0.06 (0.00009-0.07	0.68
**CD4+ TNFα+ IFNγ+**	0.02 (0.002-0.03)	0.01 (0.005-0.03)	0.78
**CD4+ PD-1+**	0.26 (0.14-0.35)	0.21 (0.09-0.36)	0.68
**CD8+ IFNγ+**	0.31 (0.23-0.99)	0.23 (0.0-0.34)	0.13
**CD8+ TNFα+ IFNγ+**	0.15 (0.12-0.86)	0.12 (0.06-0.17)	0.12
**CD8+ CD69+ TNFα+**	0.09 (0.04-0.31)	0.03 (0.01-0.099)	0.06

Columns show median and interquartile range of the percentage of spike specific cells for each set of T cell markers. Groups are compared with Kruskal-Wallis rank sum test.
