# Supplementary material for: Distinct receptor binding domain IgG thresholds predict protective host immunity across SARS-CoV-2 variants and time
Source: Nat Commun. 2023 Nov 2;14:7015. doi: 10.1038/s41467-023-42717-1 (PMC10622572; doi:10.1038/s41467-023-42717-1)
Supplement: Supplementary file 3 — Reporting Summary [file 41467_2023_42717_MOESM3_ESM.pdf]

nature portfolio

Corresponding author(s): Grace Kenny

Last updated by author(s): Sep 21, 2023

Reporting Summary

Nature Portfolio wishes to improve the reproducibility of the work that we publish. This form provides structure for consistency and transparency in reporting. For further information on Nature Portfolio policies, see our Editorial Policies and the Editorial Policy Checklist.

Statistics

For all statistical analyses, confirm that the following items are present in the figure legend, table legend, main text, or Methods section.

n/a	Confirmed	
☐	☒	The exact sample size (*n*) for each experimental group/condition, given as a discrete number and unit of measurement
☐	☒	A statement on whether measurements were taken from distinct samples or whether the same sample was measured repeatedly
☐	☒	The statistical test(s) used AND whether they are one- or two-sided<br>*Only common tests should be described solely by name; describe more complex techniques in the Methods section.*
☐	☒	A description of all covariates tested
☐	☒	A description of any assumptions or corrections, such as tests of normality and adjustment for multiple comparisons
☐	☒	A full description of the statistical parameters including central tendency (e.g. means) or other basic estimates (e.g. regression coefficient) AND variation (e.g. standard deviation) or associated estimates of uncertainty (e.g. confidence intervals)
☐	☒	For null hypothesis testing, the test statistic (e.g. *F*, *t*, *r*) with confidence intervals, effect sizes, degrees of freedom and *P* value noted<br>*Give P values as exact values whenever suitable.*
☒	☐	For Bayesian analysis, information on the choice of priors and Markov chain Monte Carlo settings
☒	☐	For hierarchical and complex designs, identification of the appropriate level for tests and full reporting of outcomes
☒	☐	Estimates of effect sizes (e.g. Cohen's *d*, Pearson's *r*), indicating how they were calculated

Our web collection on statistics for biologists contains articles on many of the points above.

Software and code

Policy information about availability of computer code

Data collection	Data for the All Ireland Infectious Diseases Cohort study were collected and stored using a REDCap database hosted at University College Dublin (v11.1.8).
Data analysis	The analyses and graphics in this analysis were made using R version 3.6.2. Receiver operating characteristic analyses were constructed using the pROC package version 1.18.0.

For manuscripts utilizing custom algorithms or software that are central to the research but not yet described in published literature, software must be made available to editors and reviewers. We strongly encourage code deposition in a community repository (e.g. GitHub). See the Nature Portfolio guidelines for submitting code & software for further information.

Data

Policy information about availability of data

All manuscripts must include a data availability statement. This statement should provide the following information, where applicable:

- Accession codes, unique identifiers, or web links for publicly available datasets
- A description of any restrictions on data availability
- For clinical datasets or third party data, please ensure that the statement adheres to our policy

Participant data from the All Ireland Infectious Diseases Cohort study can be requested from the All-Ireland Infectious Diseases Cohort Study group. Due to data protection guidelines, data and samples are accessed through standardised data access guidelines and all approved data access requests are approved by a local

ethics committee. Data from the EU-COVAT-1 study can be requested from the when the study is completed from the VACCELERATE - EU-COVAT-1 Part A Study Group. Viral isolate sequences are available at the following accession numbers: WT-B.1.177.18 (CEPHR_IE_B.177.18B.1.177.18_1220), GenBank accession ON350866; Beta (SARS-CoV-2/human/IRL/AIIDV1752/2021), GenBank: ON350868.1; Omicron-BA.5 (Pango lineage BA.5), GenBank accession OP508004.

Research involving human participants, their data, or biological material

Policy information about studies with human participants or human data. See also policy information about sex, gender (identity/presentation), and sexual orientation and race, ethnicity and racism.

Reporting on sex and gender	We report on sex, which was self-reported, but not gender in this study. The findings in this study apply to both sexes. Sex characteristics of each group are reported in this study. 156 (61%) of the 256 individuals in the primary cohorts in this study reported female sex, and 19 (38%) of the 50 individuals in the validation cohort reported female sex. Multivariable analyses considered sex as a covariate.
Reporting on race, ethnicity, or other socially relevant groupings	Race and ethnicity were not considered in these analyses.
Population characteristics	This study involved adult participants, the characteristics are described in Table 1.
Recruitment	Participants in the All Ireland Infectious Diseases cohort study were recruited from hospital settings in participating hospitals in Ireland. Participants were invited to participate during routine clinical encounters by study investigators. The validation cohort included individuals recruited through the VACCELERATE volunteer registry, which was advertised in channels including social media, vaccination clinics and targeted companies. As these studies took place in Europe, the findings may not be applicable to a global population. As participation was voluntary, self selection bias may have been introduced as participation was voluntary.
Ethics oversight	The All Ireland Infectious Diseases cohort study and these analyses were approved by the St Vincent's Hospital group Research Ethics Committee and the National Research Ethics Committee for COVID-19 in Ireland. The EU-COVAT-1 AGED trial was approved by the Ethics Committee of the Faculty of Medicine, University of Cologne, Germany.

Note that full information on the approval of the study protocol must also be provided in the manuscript.

Field-specific reporting

Please select the one below that is the best fit for your research. If you are not sure, read the appropriate sections before making your selection.

☒ Life sciences ☐ Behavioural & social sciences ☐ Ecological, evolutionary & environmental sciences

For a reference copy of the document with all sections, see nature.com/documents/nr-reporting-summary-flat.pdf

Life sciences study design

All studies must disclose on these points even when the disclosure is negative.

Sample size	Sample sizes of the groups were not predetermined, and groups were selected based on availability of samples with the chosen characteristics within each study group. This study is of a similar size or larger than many other studies examining live virus neutralising capacity.
Data exclusions	No data was excluded from this manuscript
Replication	In order to verify our main findings, namely that an RBD IgG threshold of 456 BAU/ml accurately predicts loss of protective neutralising capacity against immune escape variants, we explored the performance of this threshold in an independent validation cohort, which verified our primary findings. All antibody experiments were performed in duplicate and all viral neutralisation experiments were performed in duplicate or triplicate.
Randomization	As samples were drawn from an observational cohort study, randomization was not performed.
Blinding	Investigators were not blinded to study groups during clinical data collection, and participants from this study were allocated to groups from a larger cohort study after clinical data collection. Individuals who performed laboratory analysis were blinded to the participant characteristics.

Reporting for specific materials, systems and methods

We require information from authors about some types of materials, experimental systems and methods used in many studies. Here, indicate whether each material, system or method listed is relevant to your study. If you are not sure if a list item applies to your research, read the appropriate section before selecting a response.

Materials & experimental systems

n/a	Involved in the study
☐	☒ Antibodies
☐	☒ Eukaryotic cell lines
☒	☐ Palaeontology and archaeology
☒	☐ Animals and other organisms
☐	☒ Clinical data
☒	☐ Dual use research of concern
☒	☐ Plants

Methods

n/a	Involved in the study
☒	☐ ChIP-seq
☐	☒ Flow cytometry
☒	☐ MRI-based neuroimaging

Antibodies

Antibodies used

Serologic assay:
RBD IgG (Sino Biological, 40150-R007), final dilution 1.25µg/ml
Spike S1 IgG (Sino Biological, 40590-T620) final dilution 5µg/ml
Spike S2 IgG (Sino Biological, 40590-T62), final dilution 4µg/ml
Nucleocapsid IgG (Sino Biological, 40143-R001) final dilution 3µg/ml
Anti-Human IgG (Mesoscale Diagnostics, R32AJ-1) final dilution 1µg/ml
Anti-Rabbit IgG (Mesoscale Diagnostics, R32AB-5) final dilution 1µg/ml
Anti-Mouse IgG (Mesoscale Diagnostics, R32AC-5) final dilution 1µg/ml

Mesoscale diagnostics V-PLEX serologic assay
SULFO-TAG Anti-Human IgG Antibody (Mesoscale diagnostics, D21ADF-3) final dilution 1µg/ml

Flow based microneutralisation assay:
SARS/SARS-CoV-2 Nucleocapsid Monoclonal Antibody (E16C) (Invitrogen, MA1-7403) final dilution 1:100
Goat anti-mouse IgG2b-FITC (Santa Cruz Biotechnology, SC-2080) final dilution 1:500

Antigen specific T cell response:
CD3 Antibody, BV510; Viogreen (Miltenyi Biotec, 130-113-142) final dilution 1:100
CD4 Antibody, PEvio770 (Miltenyi Biotec, 130-113-227) final dilution 1:100
CD8 Antibody, PEvio700 (Miltenyi Biotec, 130-110-682)final dilution 1:100
CD69 Antibody, Vioblue (Miltenyi Biotec, 130-112-610) final dilution 1:100
PD-1 Antibody, BV711 (BioLegend®, 367428) final dilution 1:40
TNFα Antibody, APC (Miltenyi Biotec, 130-120-063) final dilution 1:100
IFNγ Antibody, AF488 (BioLegend® 502515) final dilution 1:40

Validation

All antibodies used have been validated by the manufacturer. Furthermore, antibodies were validated in the following manuscripts:

DOI: 10.1016/j.jim.2022.113345
RBD IgG (Sino Biological, 40150-R007), Spike S1 IgG (Sino Biological, 40590-T620), Spike S2 IgG (Sino Biological, 40590-T62), Nucleocapsid IgG (Sino Biological, 40143-R001), Anti-Human IgG (Mesoscale Diagnostics, R32AJ-1), Anti-Rabbit IgG (Mesoscale Diagnostics, R32AB-5), Anti-Mouse IgG (Mesoscale Diagnostics, R32AC-5), SULFO-TAG Anti-Human IgG Antibody (Mesoscale diagnostics, D21ADF-3)

doi: https://doi.org/10.1101/2023.01.18.23284713
SARS/SARS-CoV-2 Nucleocapsid Monoclonal Antibody (E16C) (Invitrogen, MA1-7403), Goat anti-mouse IgG2b-FITC (Santa Cruz Biotechnology, SC-2080)

https://doi.org/10.1002/oby.23526
CD3 Antibody, BV510; Viogreen (Miltenyi Biotec, 130-113-142), CD4 Antibody, PEvio770 (Miltenyi Biotec, 130-113-227), CD8 Antibody, PEvio700 (Miltenyi Biotec, 130-110-682), CD69 Antibody, Vioblue (Miltenyi Biotec, 130-112-610), PD-1 Antibody, BV711 (BioLegend®, 367428), TNFα Antibody, APC (Miltenyi Biotec, 130-120-063)
IFNγ Antibody, AF488 (BioLegend® 502515)

Eukaryotic cell lines

Policy information about cell lines and Sex and Gender in Research

Cell line source(s)

Vero E6 (VERO C1008, Vero 76, clone E6, Vero E6), were obtained from ATCC (ATCC CRL-1587)
Vero 369 E6/TMPRSS2 cells (#100978), obtained from the Centre For AIDS Reagents (CFAR) at the National Institute for Biological Standards and Control (NIBSC)

Authentication

Cells were authenticated by the provider. Additionally, cells were cultured independently from other cell lines, morphology was checked throughout culture, and only used between passage 2 and 20.

Mycoplasma contamination

All cell lines routinely tested negative for mycoplasma contamination

Commonly misidentified lines (See ICLAC register)	None

Clinical data

Policy information about clinical studies

All manuscripts should comply with the ICMJE guidelines for publication of clinical research and a completed CONSORT checklist must be included with all submissions.

Clinical trial registration	NA
Study protocol	NA
Data collection	NA
Outcomes	NA

Flow Cytometry

Plots

Confirm that:

☒ The axis labels state the marker and fluorochrome used (e.g. CD4-FITC).

☒ The axis scales are clearly visible. Include numbers along axes only for bottom left plot of group (a 'group' is an analysis of identical markers).

☒ All plots are contour plots with outliers or pseudocolor plots.

☒ A numerical value for number of cells or percentage (with statistics) is provided.

Methodology

Sample preparation	Peripheral blood mononuclear cells (PBMCs) were isolated from EDTA whole blood by centrifugation using SepMate PBMC isolation method with Lymphoprep (Stemcell technologies Inc). Cell concentration and live/dead differentiation was determined using a Muse Cell Analyzer. Isolated PBMCs were suspended in 10% dimethylsulfoxide (DMSO) 90% fetal bovine serum (FBS) cyropreservation media in 0.5ml aliquots at a concentration of 5-10 million cells/ml and stored in liquid nitrogen until analysis.
Instrument	Attune NxT Multi-Parameter Flow Cytometer (Antigen specific T cell assay), Beckmain Coulter Cytoflex S (neutralising assay)
Software	Antigen specific T cell assay analysis was performed using FlowJo v10.8.1 (FlowJo LLC, Tree Star, San Carlos, CA). Neutralising assay analysis was performed using CytExpert software (version 2.4.0.28, Beckman Coulter) and GraphPad Prism (Version 9.3.1).
Cell population abundance	Peripheral blood mononuclear cell (PBMC) concentration was determined using a Muse cell analyser prior to cryopreservation, and using the manual haemocytometer method following thawing and resuspension.
Gating strategy	Lymphocytes were identified and gated using forward and side scatter. Doublets were excluded using SSC-area and SSC-height parameters. T cells were identified by the positive expression of CD3. From the CD3 positive population, T cells were then subtyped according to their expression of surface proteins CD4 or CD8.

☒ Tick this box to confirm that a figure exemplifying the gating strategy is provided in the Supplementary Information.
